# Assessment of troglitazone induced liver toxicity in a dynamically perfused two-organ Micro-Bioreactor system

**DOI:** 10.1186/1753-6561-7-S6-P71

**Published:** 2013-12-04

**Authors:** Eva-Maria Materne, Caroline Frädrich, Reyk Horland, Silke Hoffmann, Sven Brincker, Alexandra Lorenz, Mathias Busek, Frank Sonntag, Udo Klotzbach, Roland Lauster, Uwe Marx, Ilka Wagner

**Affiliations:** 1 TU Berlin, Institute for Biotechnology, Faculty of Process Science and Engineering, Gustav-Meyer-Allee 25, 13355 Berlin, Germany; 2 Fraunhofer IWS Dresden, Winterbergstraße 28, 01277 Dresden, Germany

## Background

The ever-growing amount of new substances released to the market and the limited predictability of current *in vitro *test systems has led to an ample need for new substance testing solutions. Many drugs like troglitazone, that had to be removed from the market due to drug induced liver injury, show their toxic potential only after chronic long term exposure. But for long-term multiple dosing experiments, a controlled microenvironment is pivotal, as even minor alterations in extracellular conditions may greatly influence the cell physiology. Within our research program, we focused on the generation of a micro-engineered bioreactor, which can be dynamically perfused by an on-chip pump and combines at least two culture spaces for multi-organ applications. This circulatory systems better mimics the *in vivo *conditions of primary cell cultures and assures steadier, more quantifiable extracellular signaling to the cells.

## Materials and methods

Liver microtissues (aggregates of HepaRG+human hepatic stellate cells) and skin biopsies were cultured in separate inserts of a 96-well Transwell^® ^unit (Corning), which were hung inside the chip with the membrane fitting directly over the circuit. The tissues were cultivated either air/liquid interfaced (skin) or submerged in media (liver equivalent) for a culture period of 28 days. Exposing the tissues to troglitazone, the cultures were cultured for one day in normal medium and were, subsequently, exposed to 0 μM, 5 μM and 50 μM troglitazone, respectively for further 6 days. Application of troglitazone was repeated at 12 h intervals simultaneously with the medium change. In a further experiment co-cultures of liver and skin equivalents were cultured in a fully vascularized chip. Therefore, HDMECs isolated from human foreskin were seeded into the microfluidic channel system using a syringe. After even cell infusion inside the circuit the device was incubated in 5% CO_2 _at 37°C under static conditions for 3 h to allow the cells to attach to the channel walls. A frequency of 0.476 Hz was applied for continuous dynamic operation, after 10 days of monoculture, skin and liver tissue were added for co-cultivation for another 15 days.

## Results

**Figure 1:**
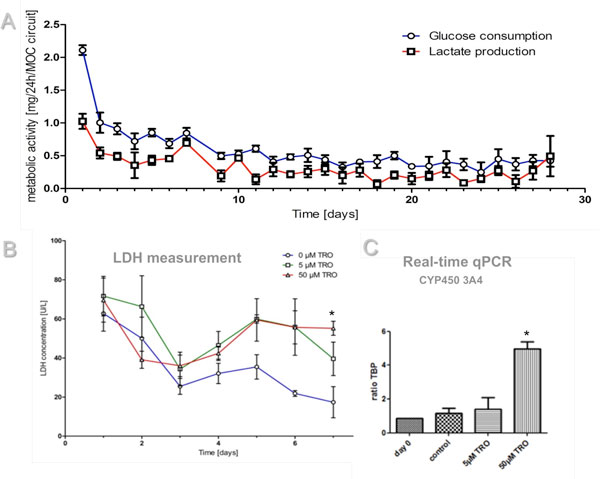
**Multi-tissue culture in the MOC device**. **(A) **Liver and skin tissue performance over 28-day MOC co-culture. Metabolic activity of the co-culture analysed in media supernatants. **(B) **LDH values **(C) **Real-time qPCR of the cytochrome P450 3A4. Statistical analysis was performed by one-way analysis of variance (ANOVA), followed by post-hoc Dunnett's pairwise multiple comparison test. * P < 0.05 versus control. Data are means ± SEM (n = 4).

Co-cultures of human artificial liver microtissues and skin biopsies have successfully proven the long-term performance of the novel microfluidic multi-organ-chip device. The metabolic activity of the co-culture analysed in media supernatants reached a steady state at day 7 of co-culture and stayed constant for the rest of the culture period (Figure 1A). Furthermore, the co-cultures revealed a dose-dependent response to a 6-day exposure to the toxic substance troglitazone. Liver microtissues showed sensitivity at different molecular levels. LDH levels measured in the media supernatants increased significantly with increasing troglitazone concentration (Figure 1B). Furthermore, an induction of Cyp450 3A4 levels on RNA level were observed (Figure 1C). In addition, a robust procedure applying pulsatile shear stress has been established to cover all fluid contact surfaces of the system with a functional, tightly closed layer of HDMECs and co-cultivation of liver, skin and endothelial cells for 15 days was successful.

## Conclusion

A unique chip-based tissue culture platform has been developed enabling the testing of drugs or chemicals on a set of miniaturized human organs. This "human-on-a-chip" platform is designed to generate high quality *in vitro *data predictive of substance safety in humans. Tissue co-cultures can be exposed to pharmaceutical substances at regimens relevant to respective guidelines, currently used for subsystemic substance testing in animals.

